# Diethyl 2,6-(2,4-dichloro­phen­yl)-4,8-dioxo-2,3,6,7-tetra­hydro-1*H*,5*H*-2,3a,4a,6,7a,8a-hexa­azacyclo­penta­[*def*]fluorene-8b,8c-dicarboxyl­ate

**DOI:** 10.1107/S1600536809005157

**Published:** 2009-03-06

**Authors:** Jiao-yang Ding, Xiao-jie Ren, Yan-ping Zhu, Neng-fang She, An-Xin Wu

**Affiliations:** a Key Laboratory of Pesticides and Chemical Biology of the Ministry of Education, College of Chemistry, Central China Normal University, Wuhan 430079, People’s Republic of China

## Abstract

The title mol­ecule, C_28_H_28_Cl_4_N_6_O_6_, is built up from four fused rings, *viz*. two nearly planar imidazole five-membered rings which adopt envelope conformations with the C=O groups at the flap position, and two triazine six-membered rings which adopt chair conformations. Each six-membered ring has a 2,4-dichloro­benzyl substituent attached to an N atom. In the mol­ecule, the two ethyl groups are each disordered between two orientations in 0.784 (16)/0.216 (16) and 0.631 (10)/0.37 (10) ratios. Weak inter­molecular C—H⋯O hydrogen bonds help to stabilize the crystal packing.

## Related literature

For the preparation of the title compound, see: Li *et al.* (2006 ▶). For general background to glycoluril and its derivatives, see: Freeman *et al.* (1981 ▶); Rebek (2005 ▶); Rowan *et al.* (1999 ▶); Wu *et al.* (2002 ▶); Cao *et al.* (2008 ▶).
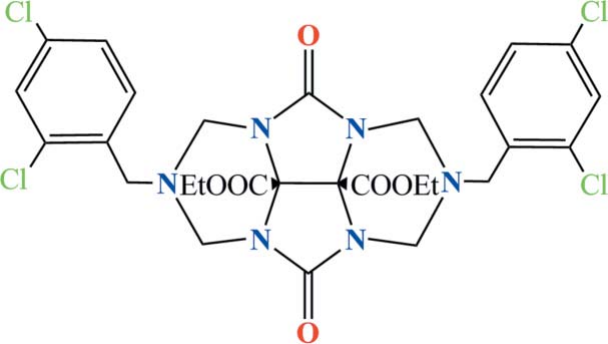


## Experimental

### 

#### Crystal data

C_28_H_28_Cl_4_N_6_O_6_
*M* *_r_* = 686.36
Monoclinic, 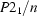

*a* = 10.0030 (11) Å
*b* = 27.1742 (15) Å
*c* = 11.2427 (2) Å
β = 93.716 (4)°
*V* = 3049.6 (4) Å^3^
*Z* = 4
Mo *K*α radiation
μ = 0.44 mm^−1^
*T* = 292 K
0.20 × 0.20 × 0.10 mm

#### Data collection

Bruker SMART 4K CCD area-detector diffractometer
Absorption correction: multi-scan (*SADABS*; Sheldrick,1996 ▶) *T*_min_ = 0.907, *T*_max_ = 0.957
24973 measured reflections
5308 independent reflections
3773 reflections with *I* > 2σ(*I*)
*R*_int_ = 0.060

#### Refinement

*R*[*F* ^2^ > 2σ(*F* ^2^)] = 0.063
*wR*(*F* ^2^) = 0.179
*S* = 1.06
5308 reflections
439 parameters
H-atom parameters constrained
Δρ_max_ = 0.47 e Å^−3^
Δρ_min_ = −0.38 e Å^−3^

### 

Data collection: *SMART* (Bruker, 2001 ▶); cell refinement: *SAINT* (Bruker, 2001 ▶); data reduction: *SAINT*; program(s) used to solve structure: *SHELXS97* (Sheldrick, 2008 ▶); program(s) used to refine structure: *SHELXL97* (Sheldrick, 2008 ▶); molecular graphics: *SHELXTL* (Sheldrick, 2008 ▶); software used to prepare material for publication: *SHELXTL*.

## Supplementary Material

Crystal structure: contains datablocks I, global. DOI: 10.1107/S1600536809005157/cv2485sup1.cif

Structure factors: contains datablocks I. DOI: 10.1107/S1600536809005157/cv2485Isup2.hkl

Additional supplementary materials:  crystallographic information; 3D view; checkCIF report

## Figures and Tables

**Table 1:** Hydrogen-bond geometry (Å, °)

*D*—H⋯*A*	*D*—H	H⋯*A*	*D*⋯*A*	*D*—H⋯*A*
C19—H19*A*⋯O2^i^	0.96	2.52	3.380 (11)	149

Symmetry code: (i) 
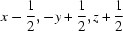
.

**Fig. 1.**
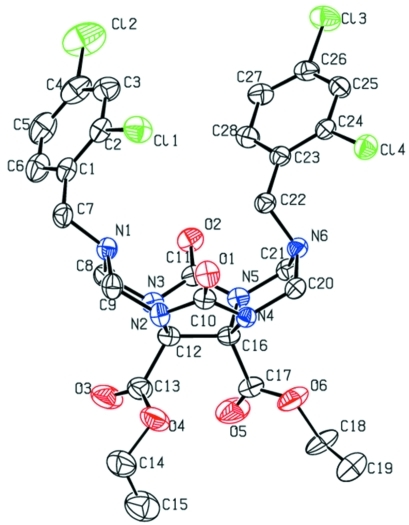
View of the molecule of (I) showing the atom-labelling scheme. Displacement ellipsoids are drawn at the 30% probability level. Only major parts of disordered atoms are shown. H atoms are represented by spheres of arbitrary radius.

## supplementary crystallographic information

### Comment

Glycoluril derivatives have been used in a variety of applications including polymer cross-linking, explosives, in the stabilization of organic compounds against photodegradation, textile waste stream purification, combinatorial chemistry, in the fields of cucurbituril chemistry and anion sensors (Freeman *et al.*, 1981; Rebek,  2005; Rowan *et al.*, 1999; Wu *et al.*, 2002). As a part of our ongoing investigation into glycoluril derivatives (Li *et al.*, 2006; Cao *et al.*, 2008), we report here the structure of the title compound (I) (Fig. 1).

The molecular structure of (I) is shown in Fig. 1. The crystal packing exhibits weak intermolecular non-classical C—H···O hydrogen bonds (Table 1).

### Experimental

The title compound was synthesized according to the known procedure (Li *et al.* (2006) in 10% isolated yield. Crystals of (I) suitable for X-ray data collection were obtained by slow evaporation of a chloroform and methaol solution in ratio of 20:1 at 298 K.

### Refinement

C-bound H atoms were initially located on a difference Fourier map, but placed in idealized positions [C–H 0.93-0.97 Å] and refined as riding, with  their U_iso_ values being set 1.2 (or 1.5 for methyl)  times of U_eq_(C). Two ethyl groups were treated as disordered between two positions each with the refined occupancies 0.784 (16)/0.216 (16) for C14-C15/C14'-C15' and 0.63 (2)/0.37 (2) for C18-C19/C18'-C19'.

### Crystal data

**Table:**

C_28_H_28_Cl_4_N_6_O_6_	*F*(000) = 1416
*M**_r_* = 686.36	*D*_x_ = 1.495 Mg m^−^^3^
Monoclinic, *P*2_1_/*n*	Mo *K*α radiation, λ = 0.71073 Å
Hall symbol: -P 2yn	Cell parameters from 7626 reflections
*a* = 10.0030 (11) Å	θ = 2.3–24.5°
*b* = 27.1742 (15) Å	µ = 0.44 mm^−^^1^
*c* = 11.2427 (2) Å	*T* = 292 K
β = 93.716 (4)°	Block, colorless
*V* = 3049.6 (4) Å^3^	0.20 × 0.20 × 0.10 mm
*Z* = 4	

### Data collection

**Table:**

Bruker SMART 4K CCD area-detector diffractometer	5308 independent reflections
Radiation source: fine-focus sealed tube	3773 reflections with *I* > 2σ(*I*)
graphite	*R*_int_ = 0.060
φ and ω scans	θ_max_ = 25.0°, θ_min_ = 1.5°
Absorption correction: multi-scan (*SADABS*; Sheldrick,1996)	*h* = −11→11
*T*_min_ = 0.907, *T*_max_ = 0.957	*k* = −32→32
24973 measured reflections	*l* = −13→13

### Refinement

**Table:**

Refinement on *F*^2^	Primary atom site location: structure-invariant direct methods
Least-squares matrix: full	Secondary atom site location: difference Fourier map
*R*[*F*^2^ > 2σ(*F*^2^)] = 0.063	Hydrogen site location: inferred from neighbouring sites
*wR*(*F*^2^) = 0.179	H-atom parameters constrained
*S* = 1.06	*w* = 1/[σ^2^(*F*_o_^2^) + (0.0927*P*)^2^ + 1.4271*P*] where *P* = (*F*_o_^2^ + 2*F*_c_^2^)/3
5308 reflections	(Δ/σ)_max_ < 0.001
439 parameters	Δρ_max_ = 0.47 e Å^−^^3^
0 restraints	Δρ_min_ = −0.38 e Å^−^^3^

### Special details

**Table:**

Geometry. All e.s.d.'s (except the e.s.d. in the dihedral angle between two l.s. planes) are estimated using the full covariance matrix. The cell e.s.d.'s are taken into account individually in the estimation of e.s.d.'s in distances, angles and torsion angles; correlations between e.s.d.'s in cell parameters are only used when they are defined by crystal symmetry. An approximate (isotropic) treatment of cell e.s.d.'s is used for estimating e.s.d.'s involving l.s. planes.
Refinement. Refinement of *F*^2^ against ALL reflections. The weighted *R*-factor *wR* and goodness of fit *S* are based on *F*^2^, conventional *R*-factors *R* are based on *F*, with *F* set to zero for negative *F*^2^. The threshold expression of *F*^2^ > σ(*F*^2^) is used only for calculating *R*-factors(gt) *etc*. and is not relevant to the choice of reflections for refinement. *R*-factors based on *F*^2^ are statistically about twice as large as those based on *F*, and *R*- factors based on ALL data will be even larger.

### Fractional atomic coordinates and isotropic or equivalent isotropic displacement parameters (Å^2^)

**Table:**

	*x*	*y*	*z*	*U*_iso_*/*U*_eq_	Occ. (<1)
C1	0.5525 (4)	0.12607 (15)	0.4170 (3)	0.0593 (9)	
C2	0.4750 (4)	0.08583 (14)	0.4444 (3)	0.0608 (10)	
C3	0.4554 (5)	0.04567 (16)	0.3692 (4)	0.0761 (12)	
H3	0.4033	0.0190	0.3893	0.091*	
C4	0.5181 (6)	0.0474 (2)	0.2614 (4)	0.0884 (15)	
C5	0.5934 (6)	0.0862 (2)	0.2305 (4)	0.0938 (16)	
H5	0.6324	0.0867	0.1577	0.113*	
C6	0.6112 (5)	0.12488 (19)	0.3089 (4)	0.0811 (13)	
H6	0.6646	0.1512	0.2885	0.097*	
C7	0.5659 (4)	0.17040 (14)	0.4962 (3)	0.0621 (10)	
H7A	0.5834	0.1601	0.5783	0.074*	
H7B	0.6408	0.1903	0.4739	0.074*	
C8	0.4272 (4)	0.22419 (13)	0.3692 (3)	0.0552 (9)	
H8A	0.4960	0.2491	0.3636	0.066*	
H8B	0.4363	0.2005	0.3055	0.066*	
C9	0.4369 (4)	0.23568 (15)	0.5801 (3)	0.0591 (9)	
H9A	0.4505	0.2197	0.6570	0.071*	
H9B	0.5070	0.2600	0.5735	0.071*	
C10	0.2018 (4)	0.24165 (12)	0.6322 (3)	0.0495 (8)	
C11	0.1878 (4)	0.21790 (12)	0.3179 (3)	0.0495 (8)	
C12	0.2615 (4)	0.27858 (12)	0.4541 (3)	0.0509 (8)	
C13	0.3251 (5)	0.33054 (15)	0.4367 (4)	0.0720 (11)	
C14	0.3744 (8)	0.4047 (2)	0.5355 (7)	0.108 (3)	0.784 (16)
H14A	0.4630	0.3988	0.5082	0.129*	0.784 (16)
H14B	0.3838	0.4158	0.6177	0.129*	0.784 (16)
C15	0.3013 (12)	0.4432 (3)	0.4579 (9)	0.121 (4)	0.784 (16)
H15A	0.3001	0.4332	0.3759	0.182*	0.784 (16)
H15B	0.3466	0.4742	0.4676	0.182*	0.784 (16)
H15C	0.2110	0.4464	0.4811	0.182*	0.784 (16)
C14'	0.274 (2)	0.4048 (4)	0.4613 (15)	0.18 (2)	0.216 (16)
H14C	0.1858	0.4180	0.4739	0.212*	0.216 (16)
H14D	0.2793	0.3989	0.3766	0.212*	0.216 (16)
C15'	0.382 (4)	0.4420 (7)	0.505 (3)	0.110 (12)	0.216 (16)
H15D	0.4687	0.4302	0.4855	0.165*	0.216 (16)
H15E	0.3809	0.4460	0.5896	0.165*	0.216 (16)
H15F	0.3650	0.4732	0.4665	0.165*	0.216 (16)
C16	0.1056 (4)	0.27423 (12)	0.4550 (3)	0.0484 (8)	
C17	0.0320 (4)	0.32152 (14)	0.4156 (3)	0.0631 (10)	
C18	−0.1608 (16)	0.3697 (5)	0.4237 (8)	0.092 (4)	0.63 (2)
H18A	−0.2273	0.3556	0.3669	0.110*	0.63 (2)
H18B	−0.1103	0.3943	0.3832	0.110*	0.63 (2)
C19	−0.2273 (14)	0.3923 (5)	0.5272 (9)	0.100 (4)	0.63 (2)
H19A	−0.2769	0.3674	0.5662	0.150*	0.63 (2)
H19B	−0.2871	0.4179	0.4986	0.150*	0.63 (2)
H19C	−0.1602	0.4058	0.5827	0.150*	0.63 (2)
C18'	−0.1345 (16)	0.3784 (5)	0.470 (3)	0.125 (10)	0.37 (2)
H18C	−0.1157	0.3957	0.3975	0.149*	0.37 (2)
H18D	−0.1047	0.3985	0.5381	0.149*	0.37 (2)
C19'	−0.2851 (13)	0.3666 (8)	0.472 (2)	0.099 (7)	0.37 (2)
H19D	−0.3091	0.3423	0.4126	0.149*	0.37 (2)
H19E	−0.3363	0.3960	0.4559	0.149*	0.37 (2)
H19F	−0.3037	0.3541	0.5492	0.149*	0.37 (2)
C20	−0.0401 (4)	0.22538 (12)	0.5846 (3)	0.0521 (8)	
H20A	−0.0375	0.2113	0.6640	0.063*	
H20B	−0.1189	0.2461	0.5750	0.063*	
C21	−0.0462 (4)	0.20626 (13)	0.3782 (3)	0.0535 (9)	
H21A	−0.1255	0.2262	0.3604	0.064*	
H21B	−0.0476	0.1795	0.3210	0.064*	
C22	0.0446 (4)	0.14521 (12)	0.5236 (3)	0.0517 (8)	
H22A	0.0502	0.1387	0.6086	0.062*	
H22B	0.1327	0.1552	0.5016	0.062*	
C23	0.0040 (4)	0.09876 (12)	0.4576 (3)	0.0497 (8)	
C24	−0.1053 (4)	0.07018 (12)	0.4858 (3)	0.0509 (8)	
C25	−0.1439 (4)	0.02843 (13)	0.4219 (3)	0.0575 (9)	
H25	−0.2183	0.0104	0.4414	0.069*	
C26	−0.0694 (4)	0.01411 (13)	0.3284 (3)	0.0606 (10)	
C27	0.0420 (4)	0.03969 (14)	0.3000 (3)	0.0647 (10)	
H27	0.0928	0.0290	0.2386	0.078*	
C28	0.0779 (4)	0.08189 (13)	0.3645 (3)	0.0610 (10)	
H28	0.1534	0.0994	0.3451	0.073*	
Cl1	0.39824 (13)	0.08382 (4)	0.57796 (9)	0.0830 (4)	
Cl2	0.4914 (2)	−0.00226 (7)	0.16389 (14)	0.1478 (8)	
Cl3	−0.12054 (14)	−0.03781 (4)	0.24604 (10)	0.0876 (4)	
Cl4	−0.19505 (11)	0.08564 (4)	0.60790 (8)	0.0677 (3)	
N1	0.4431 (3)	0.19939 (10)	0.4851 (2)	0.0510 (7)	
N2	0.3063 (3)	0.25959 (11)	0.5705 (2)	0.0512 (7)	
N3	0.2952 (3)	0.24689 (10)	0.3574 (2)	0.0501 (7)	
N4	0.0814 (3)	0.25627 (9)	0.5739 (2)	0.0459 (7)	
N5	0.0733 (3)	0.23663 (10)	0.3637 (2)	0.0490 (7)	
N6	−0.0517 (3)	0.18581 (10)	0.4971 (2)	0.0493 (7)	
O1	0.2128 (3)	0.21891 (10)	0.72513 (19)	0.0629 (7)	
O2	0.1917 (3)	0.18332 (10)	0.2502 (2)	0.0650 (7)	
O3	0.3988 (4)	0.34020 (11)	0.3645 (4)	0.1084 (13)	
O4	0.2940 (4)	0.35954 (12)	0.5261 (3)	0.0995 (11)	
O5	0.0633 (4)	0.34460 (12)	0.3319 (3)	0.1093 (13)	
O6	−0.0697 (4)	0.33056 (11)	0.4754 (3)	0.0944 (11)	

### Atomic displacement parameters (Å^2^)

**Table:**

	*U*^11^	*U*^22^	*U*^33^	*U*^12^	*U*^13^	*U*^23^
C1	0.046 (2)	0.072 (2)	0.060 (2)	0.0109 (19)	0.0016 (17)	0.0043 (17)
C2	0.053 (2)	0.072 (2)	0.0572 (19)	0.013 (2)	0.0034 (17)	0.0001 (17)
C3	0.083 (3)	0.071 (3)	0.073 (2)	0.008 (2)	−0.003 (2)	−0.006 (2)
C4	0.102 (4)	0.096 (4)	0.066 (3)	0.031 (3)	−0.007 (3)	−0.018 (2)
C5	0.091 (4)	0.123 (5)	0.069 (3)	0.027 (3)	0.026 (3)	0.001 (3)
C6	0.068 (3)	0.100 (3)	0.077 (3)	0.014 (3)	0.018 (2)	0.008 (2)
C7	0.043 (2)	0.073 (2)	0.069 (2)	0.0011 (18)	−0.0029 (17)	0.0057 (18)
C8	0.058 (2)	0.058 (2)	0.0500 (17)	−0.0012 (18)	0.0086 (16)	0.0056 (15)
C9	0.049 (2)	0.077 (2)	0.0511 (18)	−0.0035 (19)	−0.0026 (16)	−0.0068 (17)
C10	0.058 (2)	0.0524 (19)	0.0378 (15)	−0.0023 (16)	−0.0012 (15)	−0.0088 (13)
C11	0.063 (2)	0.0509 (18)	0.0347 (14)	0.0051 (17)	0.0006 (15)	0.0049 (13)
C12	0.060 (2)	0.0444 (17)	0.0491 (16)	−0.0005 (16)	0.0061 (15)	0.0001 (13)
C13	0.080 (3)	0.057 (2)	0.081 (3)	−0.004 (2)	0.027 (2)	−0.008 (2)
C14	0.106 (6)	0.079 (5)	0.140 (7)	−0.018 (5)	0.019 (5)	−0.031 (5)
C15	0.151 (12)	0.105 (7)	0.110 (7)	−0.014 (7)	0.030 (7)	0.011 (5)
C14'	0.18 (5)	0.18 (5)	0.17 (4)	0.01 (4)	0.01 (3)	0.00 (4)
C15'	0.11 (3)	0.10 (3)	0.12 (3)	0.00 (2)	0.018 (19)	−0.01 (2)
C16	0.060 (2)	0.0430 (17)	0.0423 (15)	0.0033 (16)	0.0027 (15)	0.0004 (13)
C17	0.072 (3)	0.051 (2)	0.066 (2)	0.0129 (19)	0.000 (2)	−0.0007 (18)
C18	0.113 (11)	0.081 (6)	0.081 (7)	0.060 (6)	0.003 (5)	0.018 (5)
C19	0.100 (9)	0.103 (8)	0.097 (6)	0.039 (7)	0.011 (6)	0.002 (5)
C18'	0.113 (19)	0.130 (18)	0.13 (2)	0.020 (14)	0.001 (16)	−0.001 (15)
C19'	0.097 (13)	0.106 (14)	0.095 (12)	0.011 (10)	0.004 (10)	0.026 (10)
C20	0.051 (2)	0.0533 (19)	0.0520 (17)	0.0072 (16)	0.0061 (15)	−0.0025 (14)
C21	0.050 (2)	0.0559 (19)	0.0527 (18)	0.0066 (17)	−0.0107 (16)	−0.0028 (15)
C22	0.050 (2)	0.0482 (18)	0.0563 (18)	0.0040 (15)	−0.0042 (16)	0.0011 (14)
C23	0.048 (2)	0.0466 (18)	0.0533 (17)	0.0039 (16)	−0.0074 (15)	0.0047 (14)
C24	0.046 (2)	0.0503 (18)	0.0555 (18)	0.0068 (16)	−0.0051 (15)	0.0072 (14)
C25	0.054 (2)	0.052 (2)	0.065 (2)	−0.0055 (17)	−0.0065 (18)	0.0079 (16)
C26	0.070 (3)	0.0468 (19)	0.063 (2)	0.0026 (18)	−0.0148 (19)	−0.0009 (16)
C27	0.078 (3)	0.054 (2)	0.063 (2)	0.004 (2)	0.0075 (19)	−0.0032 (17)
C28	0.065 (3)	0.054 (2)	0.064 (2)	−0.0024 (18)	0.0025 (18)	0.0014 (16)
Cl1	0.1020 (10)	0.0801 (7)	0.0695 (6)	−0.0051 (6)	0.0263 (6)	0.0059 (5)
Cl2	0.207 (2)	0.1358 (14)	0.0978 (10)	0.0362 (13)	−0.0166 (11)	−0.0540 (10)
Cl3	0.1073 (10)	0.0689 (7)	0.0841 (7)	−0.0126 (6)	−0.0125 (6)	−0.0194 (5)
Cl4	0.0647 (7)	0.0725 (6)	0.0667 (6)	0.0066 (5)	0.0095 (5)	0.0043 (4)
N1	0.0473 (18)	0.0591 (17)	0.0463 (14)	0.0018 (14)	0.0008 (12)	0.0033 (12)
N2	0.0467 (19)	0.0648 (18)	0.0421 (13)	−0.0064 (14)	0.0030 (12)	−0.0047 (12)
N3	0.059 (2)	0.0498 (15)	0.0419 (13)	0.0031 (13)	0.0039 (12)	0.0020 (11)
N4	0.0463 (18)	0.0474 (14)	0.0440 (13)	0.0013 (12)	0.0036 (12)	−0.0033 (11)
N5	0.055 (2)	0.0490 (15)	0.0422 (13)	0.0025 (13)	−0.0017 (13)	−0.0004 (11)
N6	0.0467 (18)	0.0474 (15)	0.0533 (14)	0.0034 (13)	−0.0012 (13)	−0.0005 (12)
O1	0.0677 (18)	0.0818 (17)	0.0387 (11)	−0.0032 (14)	−0.0007 (11)	0.0032 (11)
O2	0.079 (2)	0.0672 (16)	0.0484 (12)	0.0052 (14)	0.0034 (12)	−0.0155 (12)
O3	0.140 (4)	0.0620 (19)	0.132 (3)	−0.016 (2)	0.075 (3)	0.0008 (18)
O4	0.114 (3)	0.0619 (18)	0.128 (3)	−0.0258 (19)	0.047 (2)	−0.0256 (19)
O5	0.140 (3)	0.082 (2)	0.109 (2)	0.032 (2)	0.025 (2)	0.045 (2)
O6	0.110 (3)	0.074 (2)	0.101 (2)	0.047 (2)	0.026 (2)	0.0228 (17)

### Geometric parameters (Å, °)

**Table:**

C1—C6	1.384 (6)	C15'—H15D	0.9600
C1—C2	1.387 (5)	C15'—H15E	0.9600
C1—C7	1.499 (5)	C15'—H15F	0.9600
C2—C3	1.387 (5)	C16—N4	1.457 (4)
C2—Cl1	1.731 (4)	C16—N5	1.469 (4)
C3—C4	1.401 (7)	C16—C17	1.532 (5)
C3—H3	0.9300	C17—O5	1.189 (5)
C4—C5	1.354 (7)	C17—O6	1.279 (5)
C4—Cl2	1.749 (5)	C18—O6	1.494 (7)
C5—C6	1.375 (7)	C18—C19	1.508 (9)
C5—H5	0.9300	C18—H18A	0.9700
C6—H6	0.9300	C18—H18B	0.9700
C7—N1	1.458 (4)	C19—H19A	0.9600
C7—H7A	0.9700	C19—H19B	0.9600
C7—H7B	0.9700	C19—H19C	0.9600
C8—N3	1.456 (5)	C18'—O6	1.453 (10)
C8—N1	1.467 (4)	C18'—C19'	1.542 (11)
C8—H8A	0.9700	C18'—H18C	0.9700
C8—H8B	0.9700	C18'—H18D	0.9700
C9—N2	1.456 (5)	C19'—H19D	0.9600
C9—N1	1.458 (4)	C19'—H19E	0.9600
C9—H9A	0.9700	C19'—H19F	0.9600
C9—H9B	0.9700	C20—N6	1.457 (4)
C10—O1	1.213 (4)	C20—N4	1.488 (4)
C10—N2	1.381 (5)	C20—H20A	0.9700
C10—N4	1.392 (4)	C20—H20B	0.9700
C11—O2	1.212 (4)	C21—N6	1.452 (4)
C11—N3	1.382 (4)	C21—N5	1.471 (5)
C11—N5	1.383 (4)	C21—H21A	0.9700
C12—N3	1.443 (4)	C21—H21B	0.9700
C12—N2	1.450 (4)	C22—N6	1.482 (4)
C12—C16	1.566 (5)	C22—C23	1.507 (4)
C12—C13	1.566 (5)	C22—H22A	0.9700
C13—O3	1.161 (5)	C22—H22B	0.9700
C13—O4	1.330 (5)	C23—C24	1.395 (5)
C14—O4	1.467 (6)	C23—C28	1.397 (5)
C14—C15	1.518 (8)	C24—C25	1.384 (5)
C14—H14A	0.9700	C24—Cl4	1.740 (4)
C14—H14B	0.9700	C25—C26	1.382 (6)
C15—H15A	0.9600	C25—H25	0.9300
C15—H15B	0.9600	C26—C27	1.369 (6)
C15—H15C	0.9600	C26—Cl3	1.746 (4)
C14'—O4	1.438 (11)	C27—C28	1.392 (5)
C14'—C15'	1.539 (11)	C27—H27	0.9300
C14'—H14C	0.9700	C28—H28	0.9300
C14'—H14D	0.9700		
			
C6—C1—C2	116.9 (4)	O6—C17—C16	113.1 (3)
C6—C1—C7	120.9 (4)	O6—C18—C19	106.1 (6)
C2—C1—C7	122.1 (3)	O6—C18—H18A	110.5
C3—C2—C1	122.9 (4)	C19—C18—H18A	110.5
C3—C2—Cl1	116.8 (3)	O6—C18—H18B	110.5
C1—C2—Cl1	120.2 (3)	C19—C18—H18B	110.5
C2—C3—C4	116.6 (5)	H18A—C18—H18B	108.7
C2—C3—H3	121.7	O6—C18'—C19'	104.3 (9)
C4—C3—H3	121.7	O6—C18'—H18C	110.9
C5—C4—C3	122.4 (4)	C19'—C18'—H18C	110.9
C5—C4—Cl2	120.4 (4)	O6—C18'—H18D	110.9
C3—C4—Cl2	117.2 (5)	C19'—C18'—H18D	110.9
C4—C5—C6	118.8 (5)	H18C—C18'—H18D	108.9
C4—C5—H5	120.6	C18'—C19'—H19D	109.5
C6—C5—H5	120.6	C18'—C19'—H19E	109.5
C5—C6—C1	122.4 (5)	H19D—C19'—H19E	109.5
C5—C6—H6	118.8	C18'—C19'—H19F	109.5
C1—C6—H6	118.8	H19D—C19'—H19F	109.5
N1—C7—C1	109.9 (3)	H19E—C19'—H19F	109.5
N1—C7—H7A	109.7	N6—C20—N4	113.0 (3)
C1—C7—H7A	109.7	N6—C20—H20A	109.0
N1—C7—H7B	109.7	N4—C20—H20A	109.0
C1—C7—H7B	109.7	N6—C20—H20B	109.0
H7A—C7—H7B	108.2	N4—C20—H20B	109.0
N3—C8—N1	108.7 (3)	H20A—C20—H20B	107.8
N3—C8—H8A	109.9	N6—C21—N5	113.3 (2)
N1—C8—H8A	109.9	N6—C21—H21A	108.9
N3—C8—H8B	109.9	N5—C21—H21A	108.9
N1—C8—H8B	109.9	N6—C21—H21B	108.9
H8A—C8—H8B	108.3	N5—C21—H21B	108.9
N2—C9—N1	109.2 (3)	H21A—C21—H21B	107.7
N2—C9—H9A	109.8	N6—C22—C23	111.9 (2)
N1—C9—H9A	109.8	N6—C22—H22A	109.2
N2—C9—H9B	109.8	C23—C22—H22A	109.2
N1—C9—H9B	109.8	N6—C22—H22B	109.2
H9A—C9—H9B	108.3	C23—C22—H22B	109.2
O1—C10—N2	125.7 (3)	H22A—C22—H22B	107.9
O1—C10—N4	125.4 (3)	C24—C23—C28	116.7 (3)
N2—C10—N4	108.9 (3)	C24—C23—C22	123.0 (3)
O2—C11—N3	126.0 (4)	C28—C23—C22	120.3 (3)
O2—C11—N5	125.5 (3)	C25—C24—C23	122.3 (4)
N3—C11—N5	108.4 (3)	C25—C24—Cl4	117.8 (3)
N3—C12—N2	113.2 (3)	C23—C24—Cl4	119.8 (3)
N3—C12—C16	104.0 (3)	C26—C25—C24	118.7 (4)
N2—C12—C16	102.5 (3)	C26—C25—H25	120.7
N3—C12—C13	109.1 (3)	C24—C25—H25	120.7
N2—C12—C13	109.2 (3)	C27—C26—C25	121.4 (3)
C16—C12—C13	118.8 (3)	C27—C26—Cl3	120.1 (3)
O3—C13—O4	125.7 (4)	C25—C26—Cl3	118.5 (3)
O3—C13—C12	124.9 (4)	C26—C27—C28	118.9 (4)
O4—C13—C12	108.9 (4)	C26—C27—H27	120.5
O4—C14—C15	107.0 (6)	C28—C27—H27	120.5
O4—C14—H14A	110.3	C27—C28—C23	121.9 (4)
C15—C14—H14A	110.3	C27—C28—H28	119.0
O4—C14—H14B	110.3	C23—C28—H28	119.0
C15—C14—H14B	110.3	C7—N1—C9	112.3 (3)
H14A—C14—H14B	108.6	C7—N1—C8	111.5 (3)
O4—C14'—C15'	109.2 (11)	C9—N1—C8	109.4 (3)
O4—C14'—H14C	109.8	C10—N2—C12	112.3 (3)
C15'—C14'—H14C	109.8	C10—N2—C9	120.7 (3)
O4—C14'—H14D	109.8	C12—N2—C9	116.6 (3)
C15'—C14'—H14D	109.8	C11—N3—C12	111.6 (3)
H14C—C14'—H14D	108.3	C11—N3—C8	118.0 (3)
C14'—C15'—H15D	109.5	C12—N3—C8	116.0 (3)
C14'—C15'—H15E	109.5	C10—N4—C16	109.7 (3)
H15D—C15'—H15E	109.5	C10—N4—C20	119.1 (3)
C14'—C15'—H15F	109.5	C16—N4—C20	116.6 (2)
H15D—C15'—H15F	109.5	C11—N5—C16	111.5 (3)
H15E—C15'—H15F	109.5	C11—N5—C21	122.3 (3)
N4—C16—N5	111.5 (3)	C16—N5—C21	117.2 (3)
N4—C16—C17	116.2 (3)	C21—N6—C20	109.5 (3)
N5—C16—C17	107.5 (2)	C21—N6—C22	114.0 (3)
N4—C16—C12	104.8 (2)	C20—N6—C22	113.0 (2)
N5—C16—C12	102.8 (3)	C13—O4—C14'	99.0 (6)
C17—C16—C12	113.2 (3)	C13—O4—C14	113.4 (4)
O5—C17—O6	125.0 (4)	C17—O6—C18'	121.3 (13)
O5—C17—C16	121.7 (4)	C17—O6—C18	114.7 (6)
			
C6—C1—C2—C3	0.1 (6)	N3—C12—N2—C9	39.0 (4)
C7—C1—C2—C3	176.3 (3)	C16—C12—N2—C9	150.3 (3)
C6—C1—C2—Cl1	179.7 (3)	C13—C12—N2—C9	−82.8 (4)
C7—C1—C2—Cl1	−4.1 (5)	N1—C9—N2—C10	92.2 (3)
C1—C2—C3—C4	−0.2 (6)	N1—C9—N2—C12	−50.0 (4)
Cl1—C2—C3—C4	−179.8 (3)	O2—C11—N3—C12	−168.9 (3)
C2—C3—C4—C5	−0.5 (7)	N5—C11—N3—C12	14.2 (3)
C2—C3—C4—Cl2	−178.6 (3)	O2—C11—N3—C8	−30.8 (4)
C3—C4—C5—C6	1.3 (8)	N5—C11—N3—C8	152.4 (3)
Cl2—C4—C5—C6	179.3 (4)	N2—C12—N3—C11	98.8 (3)
C4—C5—C6—C1	−1.5 (7)	C16—C12—N3—C11	−11.7 (3)
C2—C1—C6—C5	0.8 (6)	C13—C12—N3—C11	−139.3 (3)
C7—C1—C6—C5	−175.5 (4)	N2—C12—N3—C8	−40.2 (4)
C6—C1—C7—N1	101.8 (4)	C16—C12—N3—C8	−150.7 (3)
C2—C1—C7—N1	−74.3 (4)	C13—C12—N3—C8	81.6 (4)
N3—C12—C13—O3	−7.4 (6)	N1—C8—N3—C11	−83.9 (3)
N2—C12—C13—O3	116.8 (5)	N1—C8—N3—C12	52.4 (4)
C16—C12—C13—O3	−126.2 (5)	O1—C10—N4—C16	−169.2 (3)
N3—C12—C13—O4	−179.9 (4)	N2—C10—N4—C16	14.1 (3)
N2—C12—C13—O4	−55.6 (5)	O1—C10—N4—C20	−31.0 (4)
C16—C12—C13—O4	61.3 (5)	N2—C10—N4—C20	152.3 (3)
N3—C12—C16—N4	121.5 (3)	N5—C16—N4—C10	99.9 (3)
N2—C12—C16—N4	3.4 (3)	C17—C16—N4—C10	−136.4 (3)
C13—C12—C16—N4	−117.1 (3)	C12—C16—N4—C10	−10.6 (3)
N3—C12—C16—N5	4.8 (3)	N5—C16—N4—C20	−39.4 (4)
N2—C12—C16—N5	−113.3 (3)	C17—C16—N4—C20	84.3 (4)
C13—C12—C16—N5	126.3 (3)	C12—C16—N4—C20	−149.9 (3)
N3—C12—C16—C17	−110.9 (3)	N6—C20—N4—C10	−86.1 (3)
N2—C12—C16—C17	131.0 (3)	N6—C20—N4—C16	49.3 (4)
C13—C12—C16—C17	10.5 (4)	O2—C11—N5—C16	172.4 (3)
N4—C16—C17—O5	165.1 (4)	N3—C11—N5—C16	−10.7 (3)
N5—C16—C17—O5	−69.3 (5)	O2—C11—N5—C21	26.5 (4)
C12—C16—C17—O5	43.6 (5)	N3—C11—N5—C21	−156.6 (2)
N4—C16—C17—O6	−20.0 (5)	N4—C16—N5—C11	−108.5 (3)
N5—C16—C17—O6	105.6 (4)	C17—C16—N5—C11	123.1 (3)
C12—C16—C17—O6	−141.5 (3)	C12—C16—N5—C11	3.3 (3)
N6—C22—C23—C24	−71.1 (4)	N4—C16—N5—C21	39.3 (4)
N6—C22—C23—C28	110.0 (4)	C17—C16—N5—C21	−89.1 (3)
C28—C23—C24—C25	−3.1 (4)	C12—C16—N5—C21	151.1 (3)
C22—C23—C24—C25	177.9 (3)	N6—C21—N5—C11	95.4 (3)
C28—C23—C24—Cl4	174.9 (2)	N6—C21—N5—C16	−48.8 (4)
C22—C23—C24—Cl4	−4.1 (4)	N5—C21—N6—C20	54.6 (4)
C23—C24—C25—C26	1.4 (5)	N5—C21—N6—C22	−73.1 (3)
Cl4—C24—C25—C26	−176.6 (2)	N4—C20—N6—C21	−54.7 (3)
C24—C25—C26—C27	1.3 (5)	N4—C20—N6—C22	73.5 (3)
C24—C25—C26—Cl3	−178.6 (2)	C23—C22—N6—C21	−72.1 (4)
C25—C26—C27—C28	−2.1 (5)	C23—C22—N6—C20	162.0 (3)
Cl3—C26—C27—C28	177.8 (3)	O3—C13—O4—C14'	45.2 (11)
C26—C27—C28—C23	0.2 (5)	C12—C13—O4—C14'	−142.4 (10)
C24—C23—C28—C27	2.3 (5)	O3—C13—O4—C14	−6.7 (8)
C22—C23—C28—C27	−178.7 (3)	C12—C13—O4—C14	165.7 (5)
C1—C7—N1—C9	165.7 (3)	C15'—C14'—O4—C13	−114.3 (17)
C1—C7—N1—C8	−71.1 (4)	C15'—C14'—O4—C14	−1.5 (17)
N2—C9—N1—C7	−174.1 (3)	C15—C14—O4—C13	89.0 (7)
N2—C9—N1—C8	61.5 (4)	C15—C14—O4—C14'	6.2 (9)
N3—C8—N1—C7	172.4 (3)	O5—C17—O6—C18'	−19.0 (14)
N3—C8—N1—C9	−62.8 (3)	C16—C17—O6—C18'	166.3 (13)
O1—C10—N2—C12	171.3 (3)	O5—C17—O6—C18	7.4 (10)
N4—C10—N2—C12	−12.0 (4)	C16—C17—O6—C18	−167.3 (8)
O1—C10—N2—C9	27.5 (5)	C19'—C18'—O6—C17	141.8 (19)
N4—C10—N2—C9	−155.7 (3)	C19'—C18'—O6—C18	61 (2)
N3—C12—N2—C10	−106.4 (3)	C19—C18—O6—C17	−152.4 (13)
C16—C12—N2—C10	5.0 (3)	C19—C18—O6—C18'	−41 (3)
C13—C12—N2—C10	131.9 (3)		

### Hydrogen-bond geometry (Å, °)

**Table:**

*D*—H···*A*	*D*—H	H···*A*	*D*···*A*	*D*—H···*A*
C19—H19A···O2^i^	0.96	2.52	3.380 (11)	149

Symmetry codes: (i) *x*−1/2, −*y*+1/2, *z*+1/2.
